# Compton Scattering of Hermite Gaussian Wave *γ* Ray

**DOI:** 10.1038/s41598-019-44120-7

**Published:** 2019-05-29

**Authors:** Tomoyuki Maruyama, Takehito Hayakawa, Toshitaka Kajino

**Affiliations:** 1 College of Bioresource Sciences, Nihon University, Fujisawa, 252-0880 Japan; 2 National Astronomical Observatory of Japan, 2-21-1 Osawa, Mitaka, Tokyo, 181-8588 Japan; 3 National Institutes for Quantum and Radiological Science and Technology, Tokai, Ibaraki, 319-1106 Japan; 4 Beihang University, School of Physics, International Research Center for Big-Bang Cosmology and Element Genesis, Beijing, 100083 China; 5 The University of Tokyo, Bunkyo-ku, Tokyo, 113-0033 Japan

**Keywords:** Quantum optics, Single photons and quantum effects

## Abstract

One of candidates for the generation mechanism of high linearly polarized *γ* rays in *γ*-ray bursts is synchrotron radiations from high energy electrons under strong magnetic fields. If this scenario is true, Hermite Gaussian (HG) wave photons, which are one of high-order Gaussian modes, are also generated by high-order harmonic radiations in strong magnetic fields. The HG wave *γ* rays propagating along the *z*-direction have quantum numbers of nodes of *n*_*x*_ and *n*_*y*_ in the *x*- and *y*-directions, respectively. We calculate the differential cross sections for Compton scattering of photons described by HG wave function in the framework of relativistic quantum mechanics. The results indicate that it is possible to identify the HG wave photon and its quantum numbers *n*_*x*_ and *n*_*y*_ and by measuring the azimuthal angle dependence of differential cross section or the energy spectra of the scattered photon as a function of the azimuthal angle.

## Introduction

Gamma-ray bursts (GRBs) are one of the most energetic explosive phenomena in the universe^1–4^. One of remarkable features for observed GBR *γ* rays is a fact that high linear polarization was observed for some *γ* rays in the energy region of several hundred keV^5,6^. The linear polarization as high as 98 ± 33% in the prompt emission of GRB 041219A was measured by the SPI telescope on board the INTEGRAL satellite^7^. The GRB polarimeter on board the IKAROS solar power sail measured the high polarization of 70 ± 22% and $84{\,}_{-28}^{+16} \%$ for GRB 110301A and 110721A, respectively^8^. As the generation mechanism for these high linear polarization *γ* rays, some scenarios have been proposed; they are synchrotron radiations from relativistic electrons under strong magnetic fields^9^ and inverse Compton scattering on low energy photons with relativistic electrons^10^. However, the generation mechanism has been an unresolved problem.

**Figure 1:**
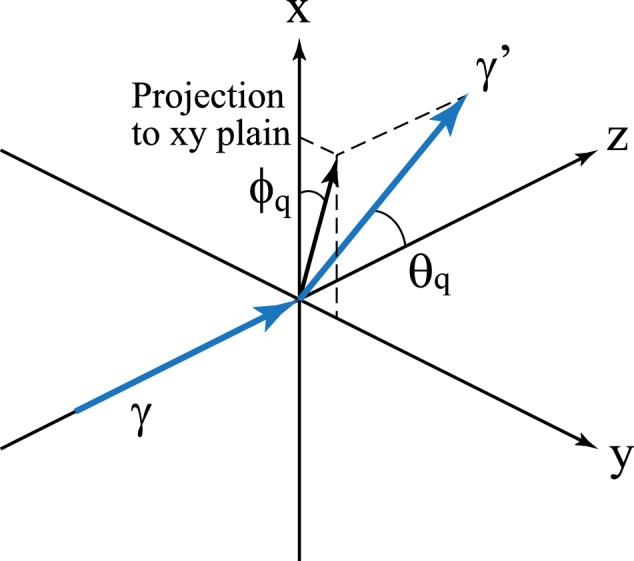
Coordinate system used in the calculation. *γ* and *γ*’ denote the initial and final photons, respectively.

**Figure 2:**
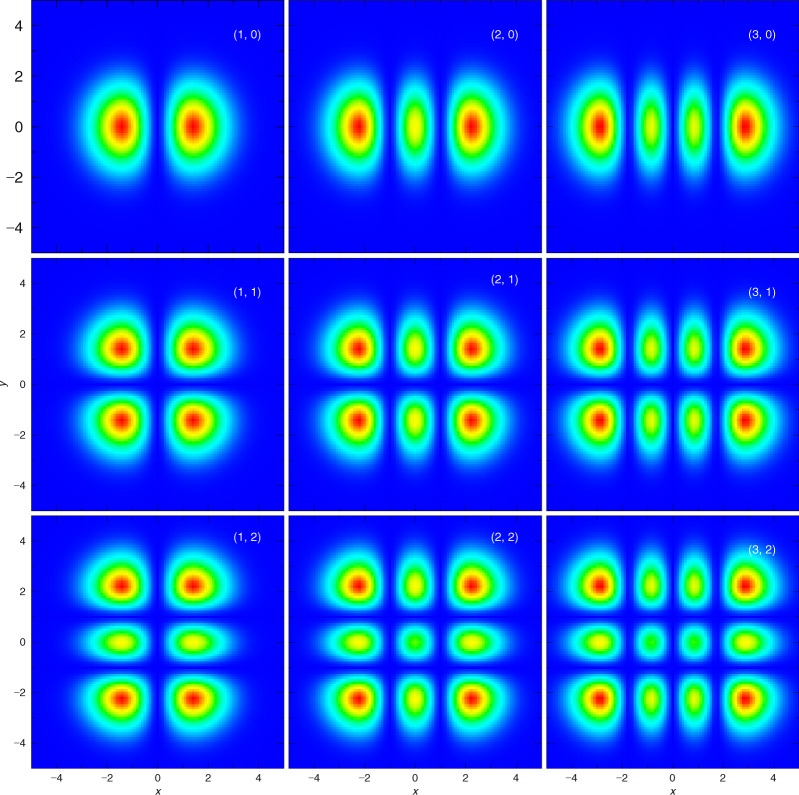
The relative strength of HG wave function at *xy*-plane, when the HG photon propagates along *z*-axis. (**a**–**i**) show the strengths for (*n*_*x*_, *n*_*y*_) = (1, 0), (2, 0), (3, 0), (1, 1), (2, 1), (3, 1), (1, 2), (2, 2), and (3, 2), respectively. Each figure shows the nodes in the strength corresponding to *n*_*x*_ and *n*_*y*_.

Here, we point out the possibility that, when high linear polarized *γ* rays are generated in GRBs, Hermite Gaussian (HG) wave *γ* rays are also generated in the same environment. The HG wave function is one of higher-order Gaussian modes of the electromagnetic field and a non-plane wave solution for Maxwell’s equation under the para-axial approximation^11^. An important feature of the HG wave photon is that the amplitude of the HG wave function has nodes of *n*_*x*_ and *n*_*y*_ in the direction of the *x*- and *y*-axes, respectively, when the photon propagates along *z*-direction (see Figs 1 and 2). This means that individual HG photons can have various quantum states at the fixed momentum, energy, and spin. Thus, the HG photons have been studied for various phenomena such as quantum entanglement, quantum communication, or quantum cryptography^12–15^. The HG wave light has been realized as a mode of laser beam in a cavity, and high power HG laser systems have been developed^16,17^.

The linearly polarized light in the energy range from ultraviolet wavelength to several hundred keV are provided by synchrotron radiations from relativistic electrons using planar undulators^18^. Each harmonic radiation including the fundamental radiation from the planar undulators has been considered to be linearly polarized. However, Sasaki & McNulty^19^ pointed out that the high-harmonic radiations from the planar undulators are the HG photon although the fundamental radiation is the linear polarization. The high-order harmonic radiations are a natural consequence of the electron motion under strong magnetic fields. Thus, when linearly polarized *γ* rays are generated by fundamental radiations under strong magnetic fields, HG photons are, in principle, also produced by high-order harmonic radiations in the same environment. Therefore, the observation of HG *γ* rays from a GRB becomes the evidence of the existence of the strong magnetic field and electron synchrotron motion.

The question that we should ask here is how identify HG *γ* rays in telescopes, because optical devices at visible and neighboring wavelengths cannot work in the several hundred keV energy region. One of the candidates to answer to this question is the use of Compton scattering. It is well known that the differential cross section with linearly polarized *γ* rays depends on the azimuthal angle between the linear polarization plane and the Compton scattered plane. Thus, the linear polarization spectrometer based upon Compton scattering has been used for the study of nuclear physics^20,21^ and for observation of astronomical *γ* rays as stated above. The Compton scattering for so-called “photon vortex”^22^ such as Laguerre Gaussian (LG) wave and Bessel wave were calculated, and the generation of the photon vortex in the universe were also discussed^23–27^. The LG wave is one of the high-order Gaussian modes and the LG (HG) wave can be represented by a linear combination of HG (LG) wave with different quantum states^11^. The Compton scattering with Bessel beams was calculated in non-relativistic^28^ and in relativistic quantum mechanics^29,30^. We previously calculated the Compton scattering with LG photons in relativistic quantum mechanics^31^. However, the differential cross section of Compton scattering with photon vortices is axial symmetric along the photon propagation direction, and hence the azimuthal angle dependence is uniform. Thus, we have proposed the coincidence measurement of the scattered photon and electron from each Compton scattering to identify the LG photon^31^.

When an initial HG wave photon propagates along the *z*-axis and its wave function has nodes of *n*_*x*_ and *n*_*y*_ in the *x*-direction and *y*-direction, respectively (see Fig. 1), the HG wave function is written as

1 $$u({\boldsymbol{r}})=\sqrt{\frac{2}{{R}_{z}}}\frac{1}{w(z)}{f}_{{n}_{x}}(\frac{\sqrt{2}x}{w(z)}){f}_{{n}_{y}}(\frac{\sqrt{2}y}{w(z)})\exp [ikz+\frac{ik{r}^{2}}{2R(z)}-i({n}_{x}+{n}_{y}+1){\theta }_{z}],$$

with

2 $$\begin{array}{rcl}{f}_{n}(x) & = & {({2}^{n}\sqrt{\pi }n!)}^{-1/2}{H}_{n}(x){e}^{-{x}^{2}/2},\\ w(z) & = & {w}_{0}\sqrt{1+\frac{{z}^{2}}{{z}_{R}^{2}}},\,R(z)=({z}^{2}+{z}_{R}^{2})/z,\,{z}_{R}=k{w}_{0}^{2}/2.\,{\theta }_{z}={\tan }^{-1}(\frac{z}{{z}_{R}}),\end{array}$$

where *k* is the energy of the initial photon, *H*_*n*_ is the *n*-th Hermite polynomial, *R*_*z*_ is the size of the system along the *z*-axis, and *w*_0_ is the waist radius at *z* = 0. The HG wave function is symmetric with respect to two planes of the *zx*- and *zy*-planes (see Fig. 2). It should be emphasized that this fact leads to a possibility that the cross section of Compton scattering of the HG photon is also symmetric with respect to these two planes. If this speculation is true, it is possible to identify the HG photon by measuring only its azimuthal angle dependence. Furthermore, one could distinguish the HG photon from the linearly polarized photon, because the Compton scattering cross section of the linearly polarized photons is only symmetric with respect to the polarization plane. In the present paper, we report the calculated differential cross section of Compton scattering of an HG photon on a rest electron in the framework of relativistic quantum mechanics. We also discuss possible measurements of HG *γ* rays in the laboratory and the universe.

## Result

We consider that a photon with a HG wave function propagates along the *z*-axis and the electron scattered in the *zx*-plane. We finally obtain the Compton scattering cross section of

3 $$\begin{array}{c}\frac{{d}^{3}\sigma }{d{\rm{\Omega }}d{E}_{q}}=\frac{{\alpha }^{2}{w}_{0}^{2}{E}_{q}}{2mk}\int \frac{d\,{\boldsymbol{p}}{}_{f}}{{E}_{f}}\delta ({E}_{f}+{E}_{q}-m-k)\delta ({Q}_{z}-\sqrt{{k}^{2}-({Q}_{x}^{2}+{Q}_{y}^{2})}){W}_{if}\\ \,\,\,\,\,\times {[{f}_{{n}_{x}}(\frac{{w}_{0}{Q}_{x}}{\sqrt{2}}){f}_{{n}_{y}}(\frac{{w}_{0}{Q}_{y}}{\sqrt{2}})]}^{2},\end{array}$$

4 $${W}_{if}=\frac{|{\boldsymbol{q}}|}{2k}+\frac{k}{2|{\boldsymbol{q}}|}-\frac{1}{2{k}^{2}}[|{{\boldsymbol{p}}}_{f}{|}^{2}-\frac{{({{\boldsymbol{p}}}_{f}\cdot {\boldsymbol{q}})}^{2}}{|{\boldsymbol{q}}{|}^{2}}],$$

with ***Q*** = ***p***_*f*_ + ***q***, where *m* is the rest mass of the electron, *q* ≡ (|***q***|, ***q***) = (*E*_*q*_, ***q***) is the momentum of the final photon, *p*_*f*_ = (*E*_*f*_, ***p***_*f*_) is the momentum of the final electron, and *α* is the fine-structure constant.

To calculate quantitatively the cross sections, we assume that HG wave photons with an energy *k* of 500 keV propagate along the *z*-axis. The HG wave function has a waist at *z* = 0 and spread beyond the waist. This spread is determined by the photon energy *k* and the waist radius *w*_0_ [see Eqs (1) and (2)]. The *w*_0_ is a free parameter in the present calculation, and depends on the generation mechanism of HG photons. We consider that the HG photons are generated by a fundamental process such as high-order harmonic radiations from high energy electrons under magnetic fields^19^, in which the waist radius probably correlates with their wave length. However, to our knowledge there is no theoretical prediction for the waist radius. The wavelength of the present assumed photon energy of 500 keV is approximately 2.48 pm. Thus, we take the waist radius *w*_0_ as 25 pm, 75 pm, and 250 pm to study the *w*_0_ dependence.

**Figure 3:**
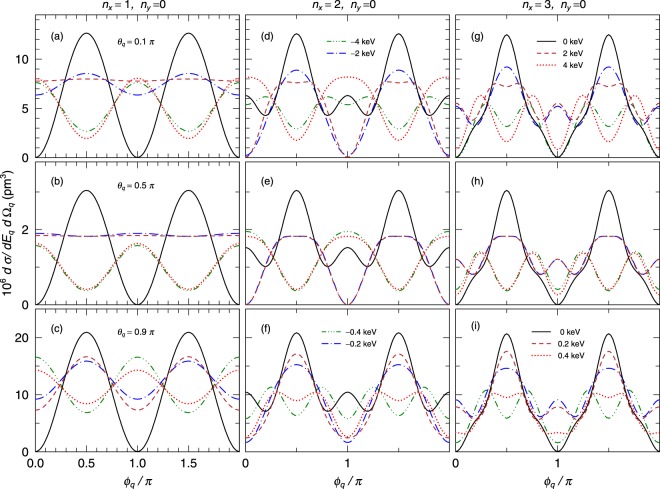
The differential cross sections of Compton scattering with HG wave function photons of the waist radius *w*_0_ = 25 pm for *n*_*x*_ = 1 and *n*_*y*_ = 0 (**a**–**c**), *n*_*x*_ = 2 and *n*_*y*_ = 0 (**d**–**f**), and *n*_*x*_ = 3 and *n*_*y*_ = 0 (**g**–**i**). The scattered polar angles are *θ*_*q*_ = 0.1*π* (**a**,**d**,**g**), *θ*_*q*_ = 0.5*π* (**b**,**e**,**h**), and *θ*_*q*_ = 0.9*π* (**c**,**f**,**i**). The solid (black), dashed (dark red), dot (red), dot-dashed (blue), two-dot-dashed (green) lines indicate the cross sections for Δ*E* = 0 keV, 2 keV, 4 keV, −2 keV, −4 keV, respectively, for *θ*_*q*_ = 0.1*π* and 0.5*π*. The solid (black), dashed (dark red), dot (red), dot-dashed (blue), two-dot-dashed (green) lines indicate the cross sections for Δ*E* = 0 keV, 0.2 keV, 0.4 keV, −0.2 keV, −0.4 keV, respectively, for *θ*_*q*_ = 0.9*π*.

Figure 3 shows the calculated differential cross sections as functions of the azimuthal angle *ϕ*_*q*_/*π* for various polar angles *θ*_*q*_ and various node numbers of *n*_*x*_ and *n*_*y*_. In the case of standard Compton scattering of a plane wave photon, the scattered photon energy, *E*_0_, is uniquely determined when the scattered angle is fixed because of the conservation law of energy and momentum. In contrast, the scattered photon energy for the incident HG wave photon may shift from that for the standard Compton scattering. Thus, we present the differential cross sections for various energy differences between the HG photon energy and the plane wave photon energy (Δ*E* = *E*_*q*_ − *E*_0_). The solid lines in Fig. 3 show the cross sections for Δ*E* = 0. In the case of *n*_*x*_ = 1 and *n*_*y*_ = 0, the *ϕ*_*q*_ dependence of the cross sections for ΔE = 0 at the three scattering polar angles of *θ*_*q*_ = 0.1*π*, 0.5*π*, and 0.9*π* are almost identical although the absolute values are different [see Fig. 3(a–c)]. This *ϕ*_*q*_ dependence is similar to that for Compton scattering with linearly polarized *γ* rays, and it is difficult to distinguish the HG photons with *n*_*x*_ = 1 and *n*_*y*_ = 0 (*n*_*x*_ = 0 and *n*_*y*_ = 1) from linear polarized photons. In contrast, in the cases of *n*_*x*_ ≥ 2, the cross sections for Δ*E* = 0 are different from that in *n*_*x*_ = 1. These results indicate that it, in principle, is possible to distinguish the HG wave photon of *n*_*x*_ ≥ 2 from the linearly polarized photons and to identify *n*_*x*_ and *n*_*y*_.

As stated above, the energy of the scattered photon may be shifted from that for the standard Compton scattering. As shown in Fig. 3, as the energy shift Δ*E* increases, the differential cross sections drastically change. Thus, it is required to determine Δ*E* in order to identify the HG photon. The range of the energy shift depends on the polar angle *θ*_*q*_ of the scattered photon; as *θ*_*q*_ increases, the energy shift decreases. The energy shift at *θ*_*q*_ = 0.5*π* is slightly lower than that at *θ*_*q*_ = 0.1*π*, but that at *θ*_*q*_ = 0.9*π* is much lower than those at *θ*_*q*_ = 0.1*π* and 0.5*π*. This indicates that by measuring the energy of the scattered photon at forward angles of *θ*_*q*_ ≤ 0.5*π*, the HG photon can be relatively easily identified. Even if the incident energy is unknown, by measuring simultaneously the deposited energy in the primary active target where Compton scattering occurs and the energy of the scattered photon in the secondary detector, it is possible to know both the energy of the incident photon and the energy shift Δ*E*. This experimental technique is being commonly used in nuclear physics and *γ*-ray astronomy.

**Figure 4:**
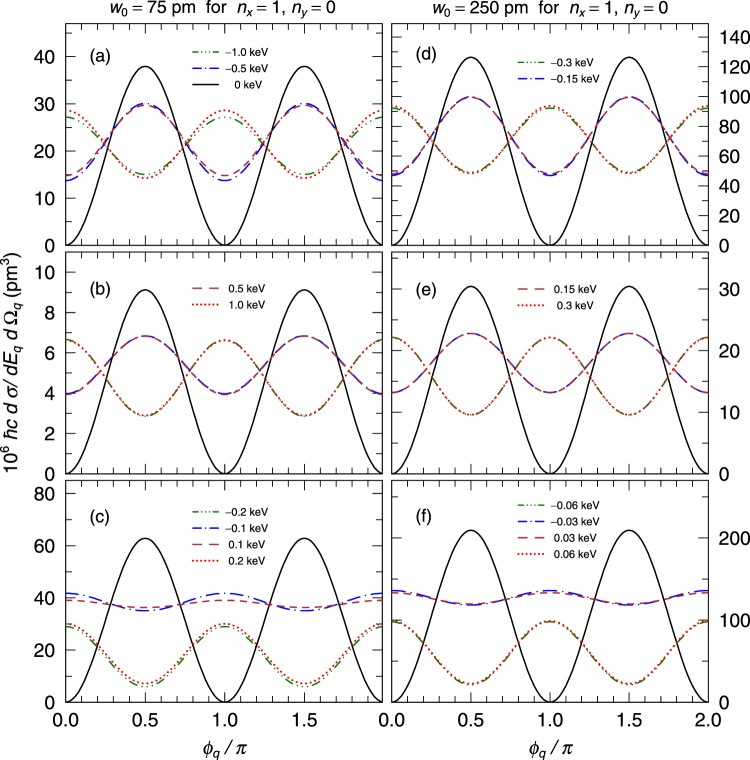
The differential cross sections of Compton scattering with HG wave function photons for *n*_*x*_ = 1 and *n*_*y*_ = 0 in the cases of the waist radius *w*_0_ = 75 pm and 250 pm. The scattered polar angles are *θ*_*q*_ = 0.1*π* (**a**,**d**), *θ*_*q*_ = 0.5*π* (**b**,**e**), and *θ*_*q*_ = 0.9*π* (**c**,**f**). The solid (black), dashed (dark red), dot (red), dot-dashed (blue), two-dot-dashed (green) lines indicate the cross sections for Δ*E* = 0 keV, 0.5 keV, 1 keV, −0.5 keV, −1 keV, respectively, for *θ*_*q*_ = 0.1*π* and 0.5*π*. The solid (black), dashed (dark red), dot (red), dot-dashed (blue), two-dot-dashed (green) lines indicate the cross sections for Δ*E* = 0 keV, 0.1 keV, 0.2 keV, −0.1 keV, −0.2 keV, respectively, for *θ*_*q*_ = 0.9*π*.

To investigate the waist radius dependence of the differential cross section, we present the cross sections for *w*_0_ = 75 pm and 250 pm in Fig. 4. We find that the results in these two cases are similar to that with *w*_0_ = 25 pm although the absolute value of the energy shift decreases as *w*_0_ increases. The energy shifts are in the range of 0.2–4 keV except for the case of *w*_0_ = 250 pm and *θ*_*q*_ = 0.9*π* (see Fig. 3). Because the typical energy resolutions of semi-conductor detectors are in several keV in full width at half maximum, the energy shifts in most of currently assumed conditions could be measured. Furthermore, even if *w*_0_ > 250 pm, because the energy shift increases with decreasing the scattered angle *θ*_*q*_, it is possible to measure the cross sections at forward angles.

**Figure 5:**
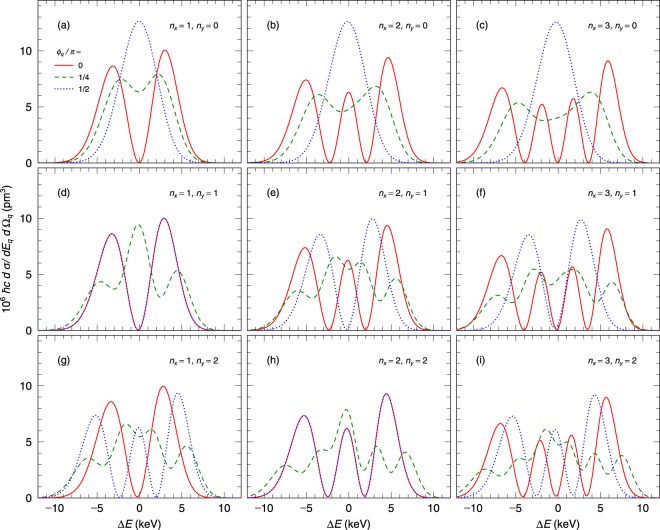
The energy spectra of the scattered photons with HG wave of the waist radius *w*_0_ = 25 pm. (**a**–**i**) show the spectra for (*n*_*x*_, *n*_*y*_) = (1, 0), (2, 0), (3, 0), (1, 1), (2, 1), (3, 1), (1, 2), (2, 2), and (3, 2), respectively. The solid (red), dashed (green), and dot (blue) lines indicate that in the case of *ϕ*_*q*_ = 0, 1/4*π*, and 1/2*π*, respectively, where *ϕ*_*q*_ is the azimuthal angle from the *zx*-plane.

Figure 5(a–c) shows the energy spectra of the photons scattered at *θ*_*q*_ = 0.1*π* for *n*_*x*_ = 1–3 and *n*_*y*_ = 0. One can see the nodes in the energy spectra at *ϕ*_*q*_ = 0 (*zx*-plane). The numbers of the nodes in Fig. 5(a–c) are 1, 2, and 3 (see the solid lines), respectively. It should be noted that each node number is identical with the corresponding *n*_*x*_. The energy spectra change as the azimuthal angle increases, and the nodes disappear at *ϕ*_*q*_ = 1/2 *π* (see the dot lines) because of *n*_*y*_ = 0. We also show the expected energy spectra in the case of *n*_*x*_ = 1–3 and *n*_*y*_ = 1 in Fig. 5(d–f). Individual energy spectra at *ϕ*_*q*_ = 0 for *n*_*x*_ = 1–3 and *n*_*y*_ = 1 [the solid lines in Fig. 5(d–f)] are identical with those in the case of *n*_*y*_ = 0 [the solid lines in Fig. 5(a–c)], where the number of the observed nodes is identical with *n*_*x*_. In contrast, the energy spectra at *ϕ*_*q*_ = 1/2 *π* for *n*_*y*_ = 1 (the dot lines) are different from those for *n*_*y*_ = 0. A single node in the energy spectra exists, corresponding to *n*_*y*_ = 1. Fig. 5(g–i) show the energy spectra for *n*_*x*_ = 1–3 and *n*_*y*_ = 2. Each energy spectrum at *ϕ*_*q*_ = 0 for *n*_*y*_ = 2 is identical with those for *n*_*y*_ = 0 and 1 (see the solid lines). The numbers of the nodes observed in three energy spectra at *ϕ*_*q*_ = 1/2 *π* are 2 corresponding to *n*_*y*_ = 2 [see the dot lines in Fig. 5(g–i)]. Note that the energy spectra at *ϕ*_*q*_ = 0 and *ϕ*_*q*_ = 1/2 *π* are identical for the cases of *n*_*x*_ = *n*_*y*_. These results show that the number of nodes observed in the energy spectrum in *zx*-plane (*ϕ*_*q*_ = 0) or *zy*-plane (*ϕ*_*q*_ = 1/2 *π*) is identical with *n*_*x*_ or *n*_*y*_. The nodes in the energy spectra originate from the nodes in the amplitude of the wave function of incident HG photons. The present calculated result indicates that the measurement of the energy spectra as functions of the azimuthal angle provides the quantum numbers *n*_*x*_ and *n*_*y*_ of the initial HG photon. As stated previously, it is difficult to identify HG photons for *n*_*x*_ = 1 and *n*_*y*_ = 0 (*n*_*x*_ = 0 and *n*_*y*_ = 1) by measuring the azimuthal angle dependence, but their identification is possible by measuring their energy spectra as shown in Fig. 5(a).

The calculated energy spectra in the energy range of ΔE < 0 and ΔE > 0 are not symmetrical to ΔE = 0 (see Fig. 5). The energy shift originates from the fact that the momenta of most HG photons are not parallel to the photon propagating direction, *z*-axis, and the angle between the incident and scattered photon momenta is different from the scattered angle from the *z*-axis, *θ*_*q*_. When the angle between the incident and scattered photon is smaller than *θ*_*q*_, the scattered photon energy increases (ΔE > 0). The differential cross section increases with decreasing the scattered angle in the forward angle region. Thus, the integrated cross section for ΔE > 0 is higher than that for ΔE < 0 as shown in Fig. 5.

## Discussion

The present results indicate that one can identify HG wave photons and their node numbers of *n*_*x*_ and *n*_*y*_ by measuring the azimuthal angle dependence of the scattering cross sections for a fixed Δ*E* or by measuring the energy spectrum of Δ*E* as a function of the azimuthal angle. A multi-segmented *γ*-ray detector system developed for polarimeters could identify HG *γ* rays from GRBs. Some polarimeters consist of two layers of segmented-detector arrays; the energy deposited by Compton scattering is measured by one of the first layer detectors and simultaneously the scattered photon is measured by one of the second layer detectors located at forward angles. This type of polarimeters are suitable for measurements of celestial HG photons because the energy shifts at forward angles are relatively large. In the previous study^31^, we concluded that the identification of LG photons is possible using the coincidence measurement of the scattered photon and electron from Compton scattering with the present radiation detectors. Because the measurement of HG photons proposed in the present study is easier comparing with the coincidence measurement of LG photons^31^, it is expected that HG *γ* rays from RGBs are measured by the next generation of satellite polarimeters.

The high linear polarization of *γ* rays from GRBs has been measured in telescopes^7,8^, and one of possible mechanisms for the polarized *γ*-ray generation is synchrotron radiations from relativistic electrons under strong magnetic fields. If this hypothesis is correct, the HG wave *γ* rays are, in principle, generated by high-order harmonic generations in the same environment, where the linear polarized *γ* rays are generated by the fundamental radiation. In contrast, in the case that the polarized *γ* rays are generated by inverse Compton scattering of relatively low energy photons with relativistic electrons, HG photons are not produced. Thus, when a *γ*-ray telescope measures HG photons from a GRB, it is the evidence that the *γ* rays are generated by synchrotron radiations under strong magnetic fields in the GRB. Because the energies of the high-order harmonic radiations are higher than the energy of the fundamental radiation by several factors, the azimuthal asymmetry originated from HG photons could be observed at energies higher than the energy at which the high linear polarization is observed.

It is expected that an HG *γ*-rays source in the laboratory is developed to examine *γ*-ray polarimeters and various experiments. We have calculated the differential Compton scattering cross section for each HG photon. Thus, the azimuthal angle dependence and the energy for individual HG photons have, in principle, the calculated pattern independent of the macro beam structure, when their *zx*- and *zy*-planes are aligned. The previous studies for light vortices presented that the vortex wave of the scattered photon is conserved after the inverse Compton scattering with relativistic electrons^30^. This suggests that the energy of a HG photon generated in GRBs may be increased by inverse Compton scattering with a relativistic electron and that it is possible to generate high energy HG photons using high power HG laser^16,17^ in the laboratory, although its detailed calculation is beyond the scope of this paper. It was proposed that HG and LG photons are generated using planar and helical undulators, respectively, with relativistic electrons by Sasaki & McNulty^19^. The generation of LG X rays has been verified using helical undulators by several groups^32,33^. Before the study by Sasaki & McNulty^19^, the generation of HG photons has been probably demonstrated using the second harmonic of a free electron laser system with a planar undulator^34^. The pattern of HG lights with *n*_*x*_ = 1 and *n*_*y*_ = 0 was observed. For *γ*-ray vortex generation, Taira *et al*.^35^ have proposed the nonlinear inverse Compton scattering with circularly polarized high peak power laser and relativistic electrons and this method has been improved by several groups^36,37^. The nonlinear Compton scattering is one of important tools to generate higher-order Gaussian mode photons, but there is no hint for the question whether the HG photon could be generated by the nonlinear inverse Compton scattering with high peak power laser.

In summary, high linearly polarized *γ* rays from *γ*-ray bursts have been measured, and one of candidates for its generation mechanism is synchrotron radiations from high energy electrons under strong magnetic fields. In this case, Hermite Gaussian (HG) wave photons are also generated by high-order harmonic radiations in strong magnetic fields. One of remarkable features for HG photons is the fact that the amplitude of the HG wave function has nodes in *x*- and *y*-directions when the HG photon propagates along the *z*-direction. We calculated the differential cross section of Compton scattering with an HG photon on a rest electron in the relativistic quantum mechanics. It is well known that the Compton scattering cross section of linearly polarized *γ* rays is symmetric with respect to the linear polarization plane. In contrast, the cross section for HG *γ* rays is symmetric with respect to two planes of the *zx*- and *zy*-planes. The present results indicate that one can identify HG wave photons and their node numbers of *n*_*x*_ and *n*_*y*_ by measuring the azimuthal angle dependence of the scattering cross sections for a fixed Δ*E* or by measuring the energy spectrum of Δ*E* as a function of the azimuthal angle. It is possible to identify the HG photon and to distinguish the HG photon from the linear polarized photon using polarimeter based upon Compton scattering except. Note that the azimuthal angle dependence for *n*_*x*_ = 1 and *n*_*y*_ = 0 (*n*_*x*_ = 0 and *n*_*y*_ = 1) is similar with that for the linear polarization but the scattered photon energy spectrum is different from it. In near future, it is expected that HG *γ* rays are measured by the next generation of satellite polarimeters. When HG photons and linearly polarized *γ* rays are measured, it gives the evidence that they are generated by synchrotron radiations in strong magnetic fields.

## Methods

We consider Compton scattering of an HG wave *γ* ray on an electron at rest, where we do not observe the electron spin and the polarization of the final photon. We choose the Lorentz Gauge and *A*_0_ = 0 for the photon field. We set the final photon wave function to be the plane wave with momentum of *q* ≡ (|***q***|, ***q***). The electron, initial photon, and final photon fields are written as

5 $$\psi (x)=\frac{1}{\sqrt{{\rm{\Omega }}}}U({\boldsymbol{p}},s){e}^{i{\rm{pr}}-i{E}_{p}t},\,{{\boldsymbol{A}}}_{i}({\boldsymbol{r}})=\frac{{\epsilon }_{i}({h}_{i})}{\sqrt{2k}}u({\boldsymbol{r}}){e}^{-ikt},\,{{\boldsymbol{A}}}_{f}({\boldsymbol{r}})=\frac{{\epsilon }_{f}({h}_{f})}{\sqrt{2|{\boldsymbol{q}}|{\rm{\Omega }}}}{e}^{i{\bf{qr}}-i|{\bf{q}}|t},$$

respectively, where Ω is the volume of the system, *U*(***p***, *s*) is the Dirac spinor of an electron with the momentum *p* = (*E*_*p*_, ***p***) and the spin *s*, and ${{\boldsymbol{\epsilon }}}_{i(f)}({h}_{i(f)})$ is the polarization vector with the helicity *h*_*i*(*f*)_. In addition, we write the initial and final momenta of the electron as *p*_*i*_ = (*m*, ***p***_*i*_) = (*m*, 0) and *p*_*f*_ = (*E*_*f*_, ***p***_*f*_), respectively. The scattering amplitude^38^ is rewritten as

6 $$\begin{array}{c}{S}_{if}=\frac{{e}^{2}}{2\sqrt{k|{\boldsymbol{q}}|{\rm{\Omega }}}}\,\bar{U}\,({{\boldsymbol{p}}}_{f},{s}_{f})[{\rlap{/}{\epsilon }}_{f}{S}_{F}({p}_{f}+q){\rlap{/}{\epsilon }}_{i}+{\rlap{/}{\epsilon }}_{i}{S}_{F}({p}_{i}-q){\rlap{/}{\epsilon }}_{f}]U({{\boldsymbol{p}}}_{i},{s}_{i})\\ \,\,\times \tilde{u}({{\boldsymbol{p}}}_{f}+{\boldsymbol{q}}-{{\boldsymbol{p}}}_{i})(2\pi )\delta ({E}_{f}+|{\boldsymbol{q}}|-m-k),\end{array}$$

with ${\epsilon }_{i,f}=(0,\,{{\boldsymbol{\epsilon }}}_{i,f})$, where *S*_*F*_ and *u*(***p***) are defined as

7 $${S}_{F}(p)=\frac{\rlap{/}{{\rm{p}}}+m}{{p}^{2}+{m}^{2}+i\delta },\tilde{u}({\boldsymbol{k}})=\int d{\boldsymbol{r}}{e}^{-i{\bf{k}}\cdot {\bf{r}}}u({\boldsymbol{r}}).$$

Then, the cross-section is given by

8 $$d\sigma =\frac{{e}^{4}}{4km}{W}_{if}{|\tilde{u}({{\boldsymbol{p}}}_{f}+{\boldsymbol{q}})|}^{2}(2\pi )\delta ({E}_{f}+|{\boldsymbol{q}}|-m-k)\frac{d{\boldsymbol{q}}\,}{{(2\pi )}^{3}|{\boldsymbol{q}}|}\frac{d{{\boldsymbol{p}}}_{f}}{{(2\pi )}^{3}{E}_{f}}$$

with

9 $${W}_{if}=\frac{m{E}_{f}}{2}\sum _{{s}_{i},{s}_{f},{h}_{f}}\,{|\bar{U}({{\boldsymbol{p}}}_{f},{s}_{f})[{\rlap{/}{\epsilon }}_{f}{S}_{F}({p}_{f}+q){\rlap{/}{\epsilon }}_{i}+{\rlap{/}{\epsilon }}_{i}{S}_{F}({p}_{i}-q){\rlap{/}{\epsilon }}_{f}]U({\bf{0}},{s}_{i})|}^{2}.$$

The Fourier transformation of *u*(***r***) in Eq. (7) becomes

10 $$\tilde{u}({\boldsymbol{Q}})=\frac{2\sqrt{2}{\pi }^{2}{w}_{0}}{\sqrt{{R}_{z}}}{f}_{{n}_{x}}[\frac{{w}_{0}{Q}_{x}}{\sqrt{2}}]{f}_{{n}_{y}}[\frac{{w}_{0}{Q}_{y}}{\sqrt{2}}]\delta ({Q}_{z}-k+\frac{{Q}_{T}^{2}}{2k}),$$

with ${Q}_{T}^{2}={Q}_{x}^{2}+{Q}_{y}^{2}$. The delta-function in Eq. (10) indicates the condition of ${Q}_{z}=k-{Q}_{T}^{2}/2k$, which is derived from the on-mass-shell condition, *k* = |***Q***|, with the para-axial approximation of *k* ≈ *Q*_*z*_ ≫ *Q*_*T*_. We improve this term by satisfying the exact kinematical condition of ${Q}_{z}=\sqrt{{k}^{2}-{Q}_{T}^{2}}$ and change Eq. (10) to

11 $$\tilde{u}({\boldsymbol{Q}})=\frac{2\sqrt{2}{\pi }^{2}{w}_{0}}{\sqrt{{R}_{z}}}{f}_{{n}_{x}}[\frac{{w}_{0}{Q}_{x}}{\sqrt{2}}]{f}_{{n}_{y}}[\frac{{w}_{0}{Q}_{y}}{\sqrt{2}}]\delta ({Q}_{z}-\sqrt{{k}^{2}-{Q}_{T}^{2}}),$$

In addition, we use the polarization vector of the initial photon to satisfy the exact relations: ${{\boldsymbol{\epsilon }}}_{i}\cdot ({{\boldsymbol{p}}}_{f}+{\boldsymbol{q}})=0$. As the result, the amplitude *W*_*if*_ becomes

12 $${W}_{if}=\frac{|{\boldsymbol{q}}|}{2k}+\frac{k}{2|{\boldsymbol{q}}|}-\frac{1}{2{k}^{2}}[|{{\boldsymbol{p}}}_{f}{|}^{2}-\frac{{({{\boldsymbol{p}}}_{f}\cdot {\boldsymbol{q}})}^{2}}{|{\boldsymbol{q}}{|}^{2}}].$$

Finally, we calculate the photon cross-section integrated over the final electron momentum.
